# Efficacy and safety of HER2 inhibitors in combination with or without pertuzumab for HER2-positive breast cancer: a systematic review and meta-analysis

**DOI:** 10.1186/s12885-019-6132-0

**Published:** 2019-10-21

**Authors:** Shanshan Chen, Yu Liang, Zhangying Feng, Mingxia Wang

**Affiliations:** Department of Clinical Pharmacology, The Fourth Hospital of Hebei Medical University and Hebei Provincial Tumor Hospital, 12 Jiankang Road, PO Box 050011, Shijiazhuang, China

**Keywords:** HER2-positive breast cancer, Pertuzumab, Trastuzumab, Trastuzumab emtansine, Dual-targeted therapy, Molecular targeted therapy

## Abstract

**Background:**

Although the dual anti-HER2 therapy, namely, pertuzumab plus trastuzumab and docetaxel, has shown promising results in HER2+ breast cancer patients, whether the dose, efficacy and safety of this treatment differs from those of other pertuzumab-based dual anti-HER2 therapies remain controversial. This systematic review evaluates the efficacy and safety of H (trastuzumab or trastuzumab emtansine ± chemotherapy) + P (pertuzumab) compared with those of H in HER2+ breast cancer patients.

**Methods:**

A comprehensive search was performed to identify eligible studies comparing the efficacy and safety of H + P versus H. The pathologic complete response (pCR), median progression-free survival (PFS) and overall survival (OS) were the primary outcomes, and safety was the secondary outcome. A subgroup analysis of pCR according to hormone receptor (HR) status was performed. All analyses were conducted using STATA 11.0.

**Results:**

Twenty-six studies (9872 patients) were identified. In the neoadjuvant setting, H + P significantly improved the pCR [odds ratio (OR) = 1.33; 95% confidence interval (CI), 1.08–1.63; *p* = 0.006]. In the metastatic setting, H + P significantly improved PFS [hazard ratios (HRs) = 0.75; 95% CI, 0.68–0.84; *p* < 0.001]. There was a trend towards better OS but that it did not reach statistical significance (HRs = 0.81; 95% CI, 0.64–1.03; *p* = 0.082). A subgroup analysis revealed that the HER2+/HR- patients who received H + P showed the highest increase in the pCR. Rash, diarrhea, epistaxis, mucosal inflammation, and anemia were significantly more frequently observed with H + P than with H, whereas myalgia was less frequent (OR = 0.91; 95% CI, 0.82–1.01; *p* = 0.072), and no significant difference in cardiac toxicity was observed between these therapies (OR = 1.26; 95% CI, 0.81–1.95; *P* = 0.309).

**Conclusions:**

Our study confirms that H + P is superior to H in the (neo)adjuvant treatment of HER2+ breast cancer, and increase the risk of acceptable and tolerable toxicity (rash, diarrhea, epistaxis, mucosal inflammation, and anemia).

**Trial registration:**

A systematic review protocol was registered with PROSPERO (identification number: CRD42018110415).

## Background

Human epidermal growth factor receptor 2 (HER2) + breast cancer is one of the most common types of breast cancer, and HER2 is amplified or overexpressed in 15 to 20% of all breast cancer patients [1]. It has been demonstrated that HER2+ breast cancer exhibits sensitivity to HER2 inhibitors, such as pertuzumab, trastuzumab, and trastuzumab emtansine. Trastuzumab (Herceptin), a humanized monoclonal antibody, was the first targeted therapy against the HER2 pathway, and its registration trial demonstrated that its combination with chemotherapy significantly improves the overall response rates and survival compared with the effects of chemotherapy alone [2]. Thus, trastuzumab has become the standard treatment for patients with HER2+ breast cancer in all treatment settings. Trastuzumab emtansine (T-DM1), an antibody-drug conjugate consisting of trastuzumab and the cytotoxic agent DM1 (derivative of maytansine), is used for the targeted delivery of cytotoxic molecules to tumors because it potentially increases efficiency and simultaneously reduces toxicity; consequently, T-DM1 has been approved by the US Food and Drug Administration (FDA) in 2013 for the treatment of HER2+ metastatic breast cancer (MBC) patients who showed progression under treatment with trastuzumab and taxane [1, 3, 4].

Although trastuzumab and T-DM1 have shown remarkable benefits in HER2+ breast cancer patients, disease resistance and intolerable toxic reactions to these drugs will invariably develop; thus, novel therapeutic approaches are needed. Significant advances in the development of new treatment combinations can offer a personalized and less aggressive approach for the management of HER2+ breast cancer patients. Pertuzumab, an HER2-targeted monoclonal antibody, inhibits ligand-dependent signaling by preventing HER2/HER3 dimerization and activates antibody-dependent cell-mediated cytotoxicity [5, 6]. Preclinical studies showed that H (trastuzumab or trastuzumab emtansine ± chemotherapy) + P (pertuzumab) is more potent and selective than either monotherapy (H). In contrast to trastuzumab/T-DM1, pertuzumab binds to a separate domain on the extracellular portion of HER2 (domain 2) and by doing so, it prevents formation of homo- and hetero-dimers which are required for activation of HER2 signaling cascade [7]. A study conducted by Cai Z et al. also strongly supports this effect [8].

Over the last decade, increasing evidence from clinical trials regarding the combinatorial use of pertuzumab has become available. The H + P combination could therefore be used to avoid drug resistance because it generates similar results in terms of pathologic complete response (pCR)/progression-free survival (PFS)/overall survival (OS) while reducing toxicity. The results from the CLEOPATRA trial [9] confirmed that the addition of pertuzumab to trastuzumab and docetaxel therapy significantly increases the PFS and OS of patients with HER2 + MBC (median PFS, 19.5 versus 12.4 months; median OS, 56.5 versus 40.8 months). The findings from phase II (NeoSphere) studies substantiate the efficacy and safety of the combination of pertuzumab with HER2-targeted therapy for patients with locally advanced, inflammatory, or early HER2+ breast cancer [10]. Patients administered pertuzumab and trastuzumab plus docetaxel exhibit a significantly improved pCR (45.8%; 95% CI, 36.1–55.7) compared with those administered trastuzumab plus docetaxel (29.0%; 95% CI, 20.6–38.5), and both groups experience a similar number of serious adverse events (AEs). According to phase Ib/IIa trials [11], the addition of pertuzumab to T-DM1 plus docetaxel results in more significant and meaningful clinical improvements in efficacy compared with the effects of T-DM1 plus docetaxel. Additionally, the results from this study showed the safety, maximum tolerated dose, and antitumor activity of the combination of pertuzumab with T-DM1 plus docetaxel in patients with HER2+ locally advanced breast cancer (LABC) or MBC.

In recent years, increasing attention has been paid to dual anti-HER2 therapies with the aim of resolving the occurrence of toxic reactions and the development of resistance. To our knowledge, no systematic analysis of H + P versus H has been reported. The present systematic review aimed to assess the efficacy and safety of H + P versus H in the (neo)adjuvant treatment of operable HER2+ breast cancer as well as metastatic disease and to stratify the other influencing factors.

## Methods

### Search strategy

The present systematic review and meta-analysis was conducted and reported according to the standards of quality detailed in the Preferred Reporting Items for Systematic Reviews and Meta-Analyses (PRISMA) statement. The present study was registered at the International Prospective Register of Systematic Reviews (registration number: CRD42018110415).

Studies were identified by searching PubMed, COCHRANE, Science Direct, EMBASE, the clinical trial registry (www.clinicaltrials.gov), and conference proceedings (American Society of Clinical Oncology, European Society of Medical Oncology, San Antonio Breast Cancer Symposium). The reference lists of key trials and review articles comparing H + P with H in the (neo)adjuvant treatment of HER2+ breast cancer were also examined to ensure that no studies were missed.

The databases were searched for studies published between 2005 (based on the first reported trial of pertuzumab efficacy in humans) and December 30, 2018. Various combinations of text and Medical Subject Headings (MeSH) terms, namely, “Breast Neoplasms OR Cancer OR Carcinomas”, “Pertuzumab OR Perjeta OR Rhumba 2C4”, “Human Epidermal Growth Factor Receptor-2 OR c-erbB-2 OR HER2-Positive”, and the following search string were used in the database searches:[“(Breast Neoplasms OR Cancer OR Carcinomas)“AND “(Pertuzumab OR Perjeta OR Rhumba 2C4)” AND“(Human Epidermal Growth Factor Receptor-2 OR c-erbB-2 OR HER2-Positive)”]. The following additional filters were included in the database search: “clinical trial”, “full text”, and “species: human”. We considered all potentially qualified studies for review, without restrictions of language or primary outcomes.

### Study selection and data extraction

Two reviewers independently screened all the publications first based on their titles and abstracts, and the studies that satisfied the inclusion criteria were then retrieved for full text assessments. Studies were included if they assessed the effectiveness and safety of H + P versus H in patients with HER2+ breast cancer, irrespective of the trial phase, the cohorts (whether prospectively or retrospectively defined), the choice of chemotherapy, and the stage of the HER2+ breast cancer patients, to improve the accuracy of our conclusions. The articles that lacked original data were excluded. If more than one publication reported results from the same trial or included the same or overlapping patient cohorts, only the outcomes from the largest and most recent publication were included.

Two independent reviewers extracted the data from the articles based on a predefined questionnaire. Any discrepancies in study selection or data extraction between reviewers were resolved by consultation with a third reviewer (Mingxia W). The following data were extracted from each study: first author’s name, year of publication, publishing journal, number of enrolled patients, neoplasm staging of patients with HER2+ breast cancer, trial phase, treatment arms, dose of HER2 inhibitors and pertuzumab, choice of chemotherapy, definition of pCR and HR status.

The main endpoints of interest with H + P were pooled to encompass the pCR, PFS, OS, and the incidence of all-grade or grade ≥ 3 AEs or cardiac toxicity (left ventricular ejection fraction (LVEF) decline < 50% or more than 10% from baseline). pCR was defined as the proportion of patients without invasive cancer in the breast and axilla (ypT0/is and ypN0) since the date of first receiving H + P or H. PFS was defined as the time of first intake of H + P or H until the time of disease progression or death from any cause. OS was defined as the interval from the initial prescription to the first occurrence of death from any cause.

### Statistical methods

For controlled trials, the hazard ratios (HRs) and 95% confidence intervals (CIs) were pooled for PFS and OS, and the number of events extracted directly from clinical trials was used to calculate the OR and 95% CI of pCR and adverse reactions. We also extracted pCR, the median PFS (in months), and the proportion of patients with adverse reactions from single-arm trials that applied H + P for the treatment of HER2+ breast cancer. Immature and interim PFS results were not included in the analysis.

The heterogeneity in the results of the studies was evaluated both visually through forest plots and *p* values and using the I-squared (I^2^) parameter, which represents the percentage of total variation across studies that is attributable to heterogeneity rather than to chance. *P* values ≤0.05 were considered significant for heterogeneity, I^2^ < 25% was considered to indicate a low level of heterogeneity and I^2^ > 75% was considered to indicate a high level of heterogeneity. If statistically significant heterogeneity was observed (I^2^ ≥ 50%), a pooled effect was calculated using a random-effect model; otherwise, a fixed-effect model was employed (I^2^ ≤ 50%). A sensitivity analysis was performed by recalculating the pooled outcome estimates after excluding each study one at a time (leave-one-out procedure). The publication bias was evaluated using both Begg’s and Egger’s tests. The quality of the eligible studies was assessed using the Cochrane Handbook for Systematic Reviews of Interventions [12]. All analyses were conducted with STATA 11.0 (State Corporation, Lake Way, Texas, USA). All tests were two-sided, and statistical significance was defined as *P* < 0.05.

### Subgroup analysis

Because the evaluation of biomarkers is highly recommended for the optimal management and decisions of the treatment of breast cancer patients, we divided the patients into two groups according to their HR status (estrogen and/or progesterone receptor positive or negative) to assess the influence of the HR status on the activity of H + P and H. Data on the influence of the HR status on outcomes were lacking in the trials included in the present study; hence, we only analyzed the differences in pCR depending on the HR status.

## Results

### Characteristics of the included studies

**Fig. 1:**
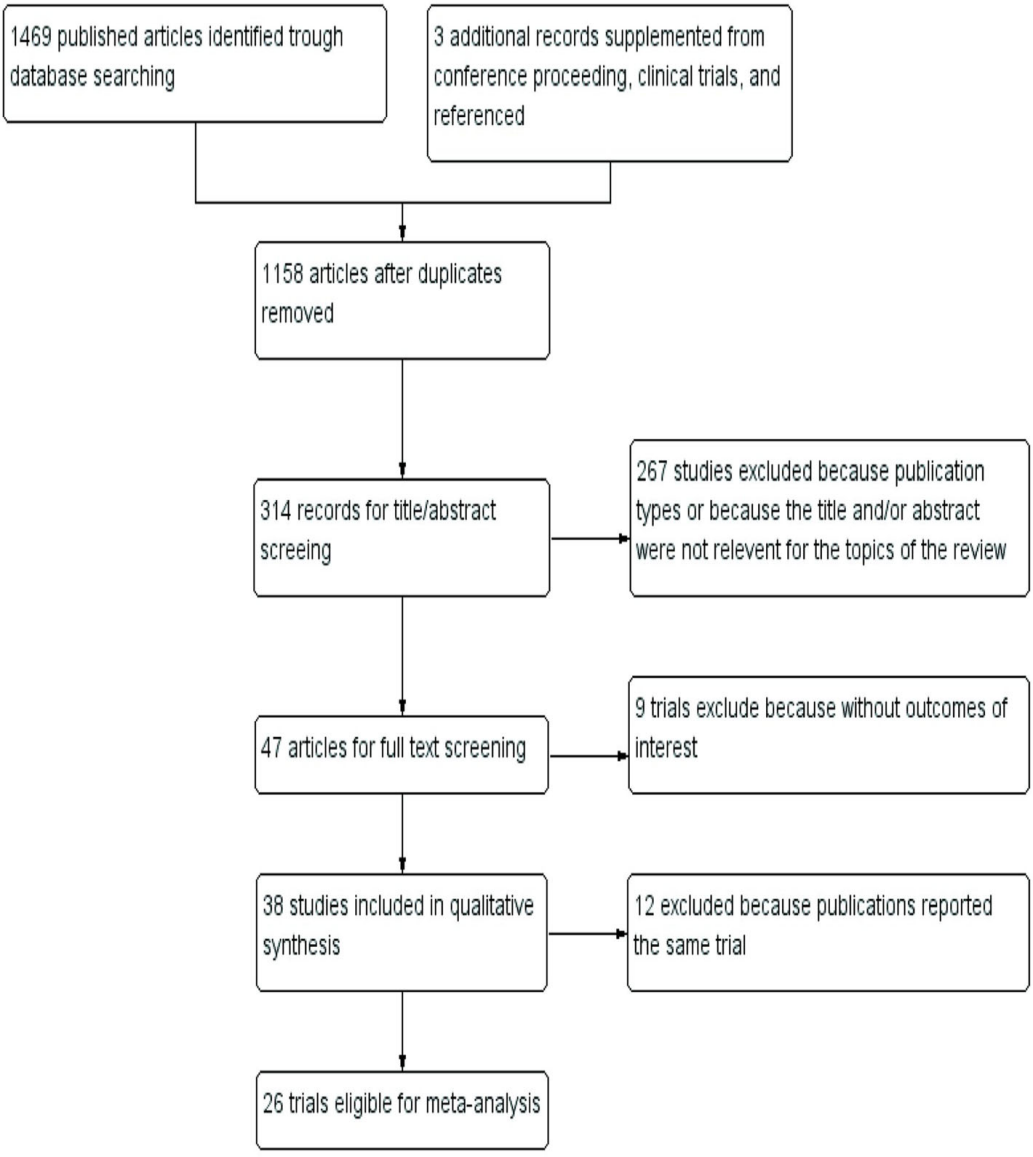
Flow diagram of the trial search and selection process

The systematic review process yielded 1469 studies limited to clinical trials from PubMed, COCHRANE, Science Direct, EMBASE, and Clinical Trials.gov, and the screening of the titles and abstracts revealed that 1422 of these articles did not match the eligibility criteria. An additional 21 studies were excluded because they were duplicates or did not describe outcomes of interest (pCR, PFS, OS, or outcomes of AEs). One additional article was included after a search of the American Society of Clinical Oncology 2016 Annual Meeting abstracts, and two articles were included after an examination of the reference lists of the included studies [9, 13, 14]. Therefore, the remaining 26 reports, which included 9872 HER2+ breast cancer patients, were investigated in the present study [9–11, 13–35]. The PRISMA flow diagram detailing the inclusion and exclusion of publications is shown in Fig. 1. The studies included in our review were published or presented from 2005 to 2018. Of these 26 studies, the 14 single-arm trials with 1098 patients included 13 studies describing pertuzumab combined with trastuzumab for the treatment of HER2+ breast cancer patients [14, 22–33] and one study describing pertuzumab combined with T-DM1 for the treatment of HER2+ breast cancer patients [34], and the 12 controlled trials with 8774 participants (4015 patients and 4759 patients in the experimental and control arms, respectively) included seven studies describing the treatment of patients with pertuzumab combined with trastuzumab versus trastuzumab alone [9, 10, 15–20, 35] and four studies describing the treatment of patients with pertuzumab combined with T-DM1 versus T-DM1 alone [11, 13, 20–22]. Moreover, pCR was reported in four controlled studies and four single-arm studies, the median PFS was reported in five controlled studies and nine single-arm studies, and OS was reported in four controlled studies. The main characteristics of the eligible studies are summarized in Table 1. The results of the quality assessments of the included studies are shown in Table 2.

**Table 1:** Characteristics of the included studies

Study	Phase	Treatment status	HER-2 therapy	Pts no.	Dosage	Chemotherapy	Efficacy endpoint	Patients status
Luca Gianni 2018 [22]	2	Neoadjuvant	P + T	30	840 mg → 420 mg q3w + 8 mg/kg → 6 mg/kg q3w	Palbociclib, Fulvestrant	pCR safety	Unilateral invasive, HER2-positivebreast cancer
Julia Foldi 2017 [23]	2	Neoadjuvant	P + T	48	During weeks 1–12, 840 mg → 420 mg q3w + 4 mg/kg → 2 mg/Kg weekly; During weeks 13–24, 420 mg + 6 mg/kg q3w;	Paclitaxel, FEC	pCR safety	stage I–III, HER2-positive invasive breast cancer
JASMEET C. SINGH 2017 [24]	retrospective study	Neoadjuvant	P + T	57	840 mg → 420 mg q3w + 8 mg/kg → 6 mg/kg q3w	AC, Paclitaxel	pCR	operable breast cancer (53) locally advanced disease(3) inflammatory breast cancer(1)
Shruti R. Tiwari 2016 [25]	retrospective study	Neoadjuvant	P + T	70	840 mg → 420 mg q3w + 8 mg/kg → 6 mg/kg q3w	Docetaxel, Carboplatin	pCR safety	I(6), II(48), and III(16), HER2-positive breast cancer
MICHAEL ANDERSSON 2017 [26]	2	Metastatic	P + T	107	co-infusion of 840 mg → 420 mg q3w + 8 mg/kg → 6 mg/kg q3w	Vinorelbine	PFS safety	HER2-positive MBC/LABC
Edith A. Perez 2016 [27]	2	Metastatic	P + T	106	Infusion of 840 mg → 420 mg q3w + 8 mg/kg → 6 mg/kg q3w, respectively	Vinorelbine	PFS safety	HER2-positive MBC/LABC
Chau Dang 2015 [28]	2	Metastatic	P + T	69	840 mg → 420 mg q3w + 8 mg/kg → 6 mg/kg q3w	Docetaxel	PFS safety	HER2-positive MBC
Bao D Dao 2015 [29]	retrospective study	Metastatic	P + T	19	NK	Taxane	PFS	HER2-positive MBC
Kazuhiro Araki 2017 [14]	2	Metastatic	P + T	30	840 mg → 420 mg q3w + 8 mg/kg → 6 mg/kg q3w	Eribulin	PFS safety	HER2-positive ABC
Jose´ Baselga 2010 [30]	2	Metastatic	P + T	66	840 mg → 420 mg q3w + 4 mg/kg → 2 mg/kg weekly or 8 mg/kg → 6 mg/kg q3w	NO	PFS safety	HER2-positive MBC
Chia C. Portera 2008 [31]	1	Metastatic	P + T	11	840 mg → 420 mg q3w + 8 mg/kg → 6 mg/kg q3w	NO	safety	HER2-positive MBC
Nicholas J. Robert 2017 [32]	retrospective study	Metastatic	P + T	266	NK	Taxane	PFS safety	HER2-positive MBC
Sabino De Placido 2018 [33]	retrospective study	Metastatic	P + T	155	840 mg → 420 mg q3w + 8 mg/kg → 6 mg/kg q3w	Taxane	PFS safety	HER2-positive MBC
Kathy D. Miller 2014 [34]	Ib/IIa	Metastatic	P + T-DM1	64	840 mg → 420 mg q3w + 3.6 mg/kg q3w	NO	PFS safety	HER2-positive MBC/LABC
Peter Beitsch 2017 [10]	prospective	Neoadjuvant	A:P + T B:T	119 178	NK	Docetaxel, Carboplatin	pCR	T4 or inflammatory HER2-positive breast cancer
Luca Gianni^a^ 2012 [15]	2	Neoadjuvant	A:P + T B:T	107 107	840 mg → 420 mg q3w + 8 mg/kg → 6 mg/kg q3w 8 mg/kg → 6 mg/kg q3w	Docetaxel	pCR safety	locally advanced, inflammatory, or early-stage HER2-positive breast cancer
Gunter von Minckwitz 2017 [16]	prospective	Adjuvant	A:P + T B:T	2400 2405	840 mg → 420 mg q3w + 8 mg/kg → 6 mg/kg q3w 8 mg/kg → 6 mg/kg q3w	FEC, Docetaxel or Paclitaxel, Carboplatin	safety	HER2-Positive EBC
Rashmi K. Murthy 2018 [17]	retrospective study	Neoadjuvant	A:P + T B:T	170 807	840 mg → 420 mg q3w + 8 mg/kg → 6 mg/kg q3w 8 mg/kg → 6 mg/kg q3w or 4 mg/kg → 2 mg/kg weekly	Paclitaxel	pCR	Stage II-III,HER-2-positive Breast Cancer
M. Martin 2016 [13]	I b /IIa	Metastatic	A:P + T-DM1 B:T-DM1	33 40	840 mg → 420 mg q3w + 3.6 mg/kg q3w 3.6 mg/kg q3w	Docetaxel	pCR safety	HER2-positive MBC/LABC
Mothaffar Rimawi 2017 [18]	2	Metastatic	A:P + T B:T	129 129	840 mg → 420 mg q3w + 8 mg/kg → 6 mg/kg q3w 8 mg/kg → 6 mg/kg q3w	AI	PFS safety	HER2-positive MBC/LABC
Ander Urruticoechea^a^ 2017 [9]	3	Metastatic	A:P + T B:T	228 224	840 mg → 420 mg q3w + 8 mg/kg → 6 mg/kg q3w 8 mg/kg → 6 mg/kg q3w	Carboplatin	PFS safety	HER2-positive MBC
Sandra M. Swain^a^ 2015 [19]	3	Metastatic	A:P + T B:T	402 406	840 mg → 420 mg q3w + 8 mg/kg → 6 mg/kg q3w 8 mg/kg → 6 mg/kg q3w	Docetaxel	PFS safety	HER2-positive MBC
Ian E. Krop^a^ 2016 [20]	I b /IIa	Metastatic	A:P + T-DM1 B:T-DM1	22 22	840 mg → 420 mg q3w + 3.6 mg/kg q3w or 2.4 mg/kg weekly 3.6 mg/kg q3w or 2.4 mg/kg weekly	Paclitaxel	PFS safety	HER2-positive MBC/LABC
Edith A. Perez^a^ 2017 [21]	3	Metastatic	A:P + T-DM1 B:T-DM1	363 367	840 mg → 420 mg q3w + 3.6 mg/kg q3w 3.6 mg/kg q3w	NO	PFS safety	HER2-positive MBC/LABC
Manish Gupta 2013 [11]	2	Metastatic	A:P + T-DM1 B:T-DM1	20 51	840 mg → 420 mg q3w + 3.6 mg/kg q3w 3.6 mg/kg q3w	NO	PFS safety	HER2-positive MBC/LABC
Nadia Hussain 2018 [35]	retrospective study	Neoadjuvant	A:P + T B:T	22 23	840 mg → 420 mg q3w + 8 mg/kg → 6 mg/kg q3w 8 mg/kg → 6 mg/kg q3w	Docetaxel, Carboplatin	safety	stages 1–3 HER2-positive breast cancer

*Abbreviations: T* Trastuzumab, *P* Pertuzumab, *T-DM1* Trastuzumab emtansine, *AC* Doxorubicin, Cyclophosphamide, *FEC* Fluorouracil (5FU), Epirubicin, and Cyclophosphamide, *AI* Aromatase Inhibitor, *pts* no patients number, *mg* milligram, *kg* kilogram, q3w three-weekly, *NK* unknown, *NO* without chemotherapy, *ABC* Advanced Breast Cancer, *MBC* Metastatic Breast Cancer, *LABC* Locally Advanced Breast Cancer, *EBC* Early Breast Cancer, *HER2* Human Epidermal Growth Factor Receptor 2

^a^ randomized controlled trials

**Table 2:** Quality assessment of included studies

Study	Random sequence generation	Allocation concealment	Blinding of participants and personnel	Blinding of outcome assessment	Incomplete outcome data	Selective reporting	Bias from other resources
Shruti R. Tiwari 2016 [25]	Low risk	Unclear	Unclear	Low risk	Low risk	Low risk	Low risk
Sandra M.Swain 2015 [19]	Low risk	Low risk	Low risk	Low risk	Low risk	Low risk	Low risk
Sabino De Placido 2018 [33]	Low risk	High risk	Unclear	Low risk	Low risk	Low risk	Low risk
Rashmi K. Murthy 2018 [17]	Low risk	Unclear	Low risk	Low risk	High risk	Low risk	Low risk
Peter Beitsch 2017 [10]	Low risk	Low risk	Low risk	Low risk	Low risk	Low risk	Low risk
Nicholas J. Robert 2017 [32]	Low risk	Unclear	Unclear	Low risk	Low risk	Low risk	Unclear
Nadia Hussain 2018 [35]	Unclear	Unclear	Unclear	Low risk	Low risk	Low risk	Low risk
Mothaffar Rimawi 2017 [18]	Low risk	Unclear	Low risk	Low risk	Low risk	Low risk	Low risk
Andersson M 2017 [26]	Low risk	Unclear	Low risk	Low risk	Low risk	Low risk	Low risk
Manish Gupta 2013 [11]	High risk	Low risk	Low risk	Low risk	High risk	High risk	Unclear
M. Martin 2016 [13]	High risk	Unclear	Low risk	Low risk	Low risk	Low risk	Low risk
Luca Gianni 2018 [22]	Low risk	Unclear	Low risk	Low risk	Low risk	Low risk	High risk
Luca Gianni 2012 [15]	Low risk	Low risk	Low risk	Low risk	Low risk	Low risk	Low risk
Kazuhiro Araki 2017 [14]	Low risk	Low risk	Unclear	Unclear	Low risk	Low risk	High risk
Kathy D. Miller 2014 [34]	Low risk	Low risk	Low risk	Low risk	Low risk	Low risk	Low risk
Julia Foldi 2017 [23]	Low risk	Low risk	Low risk	Low risk	Low risk	Low risk	Low risk
José Baselga 2010 [30]	Low risk	Low risk	Low risk	Low risk	Low risk	Low risk	Low risk
JASMEET C. SINGH 2017 [24]	Unclear	Unclear	Low risk	Low risk	Low risk	Low risk	Unclear
Ian E.Krop 2016 [20]	Low risk	Unclear	Low risk	Low risk	Low risk	High risk	Low risk
Gunter von Minckwitz 2017 [16]	Low risk	Low risk	Low risk	Low risk	Low risk	Low risk	Low risk
Edith A. Perez 2017 [21]	Low risk	Low risk	Low risk	Low risk	Low risk	Low risk	Low risk
Edith A. Perez 2016 [27]	Low risk	Unclear	Low risk	Low risk	Low risk	Low risk	Low risk
Chia C. Portera 2008 [31]	Low risk	Low risk	Low risk	Low risk	Low risk	Low risk	Low risk
Chau Dang 2015 [28]	Low risk	Unclear	Low risk	Low risk	Low risk	Low risk	Low risk
Bao D Dao 2015 [29]	Unclear	Unclear	Low risk	Low risk	Low risk	Low risk	Unclear
Ander Urruticoechea 2017 [9]	Low risk	Low risk	Low risk	Low risk	Low risk	Low risk	Low risk

### Primary outcomes

#### pCR in neoadjuvant studies and subgroup analysis

**Fig. 2:**
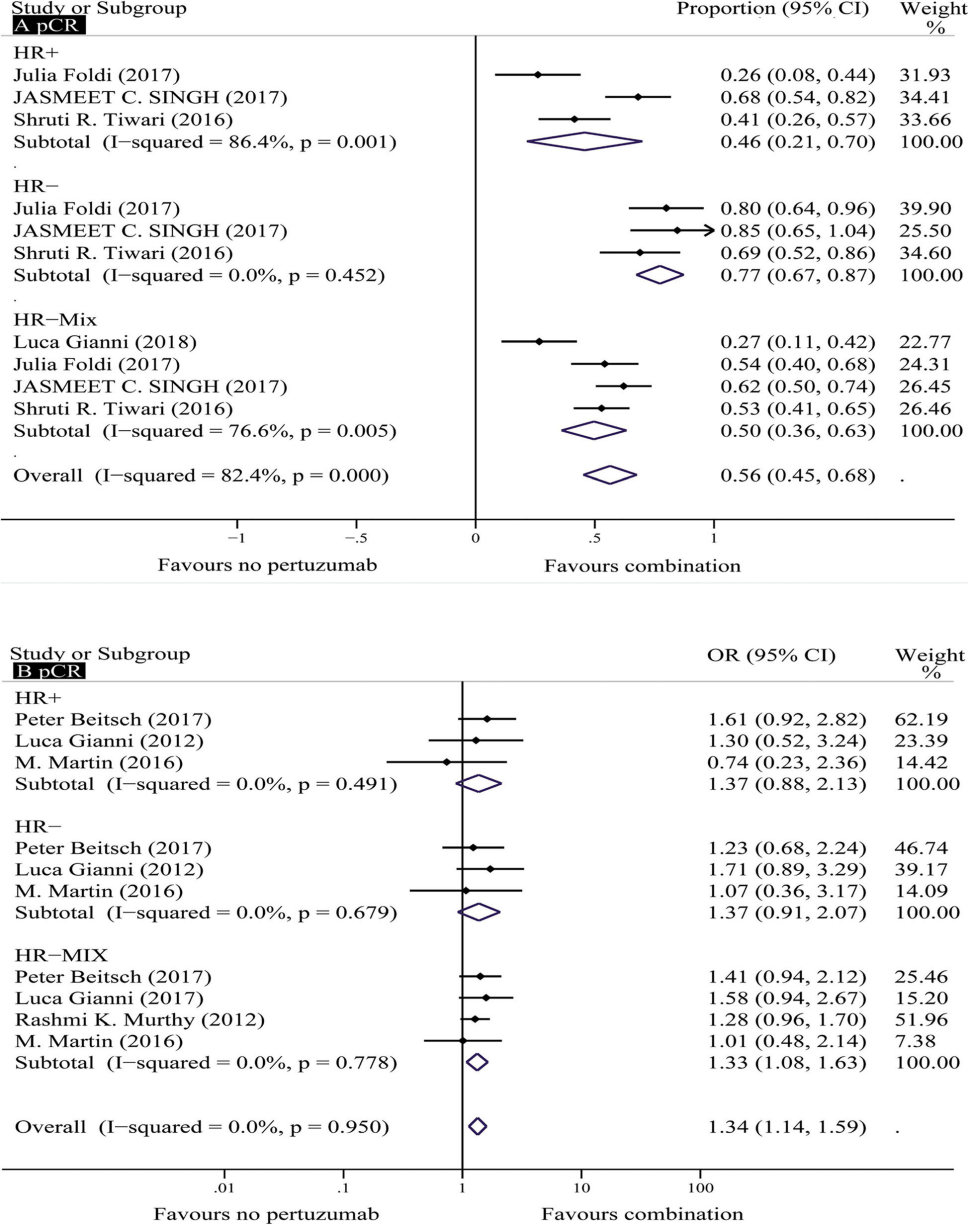
Forest plots of the pCR rates in single-arm studies (only one treatment group) (**a**): combination of pertuzumab with HER2 inhibitors for patients with HER2+ breast cancer; forest plots of the pCR rates in controlled studies (two treatment groups) (**b**): combination of pertuzumab with HER2 inhibitors versus HER2-targeted therapies without pertuzumab for patients with HER2+ breast cancer. CI = confidence interval; HER2 = human epidermal growth factor receptor 2, HR+ = hormone receptor positive, HR- = hormone receptor negative, pCR = pathologically complete response

Four single-arm trials that included 205 patients were analyzed for the pCR rate in stage Ӏ-III HER2+ breast cancer patients treated with neoadjuvant H + P [10, 13, 15, 17]. The pCR rates ranged from 0.27 to 0.62 in the four studies, and the pooled results using a random effects model showed that the absolute pCR rate was 0.56 (95% CI, 0.45–0.63). Significant heterogeneity was observed (I^2^ = 82.4%; *P* < 0.001) (Fig. 2a). In the sensitivity analysis, the estimated absolute rate equaled 0.59 (95% CI, 0.36–0.63) after removing the studies conducted by Luca Gianni and Jasmeet C. Singh.

Four controlled trials including 1448 patients (*n* = 383 in the experimental H + P groups and *n* = 1065 in the control H groups) were analyzed for the pCR rate in stage Ӏ-III HER2+ breast cancer patients [22–25]. The pooled results using a fixed-effects model demonstrated that the pCR rate of the H + P group was significantly higher than that of the H group (OR = 1.33; 95% CI, 1.08–1.63; *P* = 0.006) (Fig. 2b). Low heterogeneity was found among the included individual studies (I2 = 0.0%; *P* = 0.78), and no publication bias was not detected using Begg’s test (*P* = 0.734) and Egger’s test (*P* = 0.80). Moreover, the absolute pCR rates of the H + P and H groups were estimated to equal 55 and 44%, respectively.

A subgroup analysis based on the HR was conducted. The analysis of pCR outcomes stratified by HR status revealed that the HR status contributes to the difference in efficacy between H + P and H. A subgroup analysis of the four single-arm trials showed that the efficacy of H + P in HR- (pCR rate range, 0.69–0.85; absolute rate = 0.77; 95% CI, 0.67–0.87; *P* < 0.001) was more significant than that in HR+ (pCR rate range, 0.26–0.68; absolute rate = 0.46; 95% CI, 0.21–0.70; P < 0.001). Significant heterogeneity was observed in the HR+ group (I^2^ = 86.4%; *P* = 0.001) (Fig. 2a). The sensitivity analysis yielded an estimated absolute rate of 0.35 (95% CI, 0.21–0.70) after sequential exclusion of the study conducted by Jasmeet C. Singh. The subgroup analysis based on HR was performed in three studies, the results of the benefit ratio showed that there was a trend towards better pCR of HR- patients treated with H + P compared to that of HR+ patients [absolute rate (HR-) = 0.68; absolute rate (HR+) = 0.39]. However, the results of comparison between group H + P and group H on the efficacy of HR+/HR- breast cancer patients showed that the efficacy of H + P was not significantly better than that of H in HR+ (absolute rate = 0.39 versus 0.30) or HR- (absolute rate = 0.68 versus 0.51) breast cancer patients, and the pooled estimates using a fixed-effects model indicated no significant difference between HR+ (OR = 1.37; 95% CI, 0.88–2.13; *P* = 0.162) and HR- (OR = 1.37; 95% CI, 0.91–2.07; *P* = 0.126) breast cancer patients (Fig. 2b).

#### PFS and OS in metastatic studies or settings

**Fig. 3:**
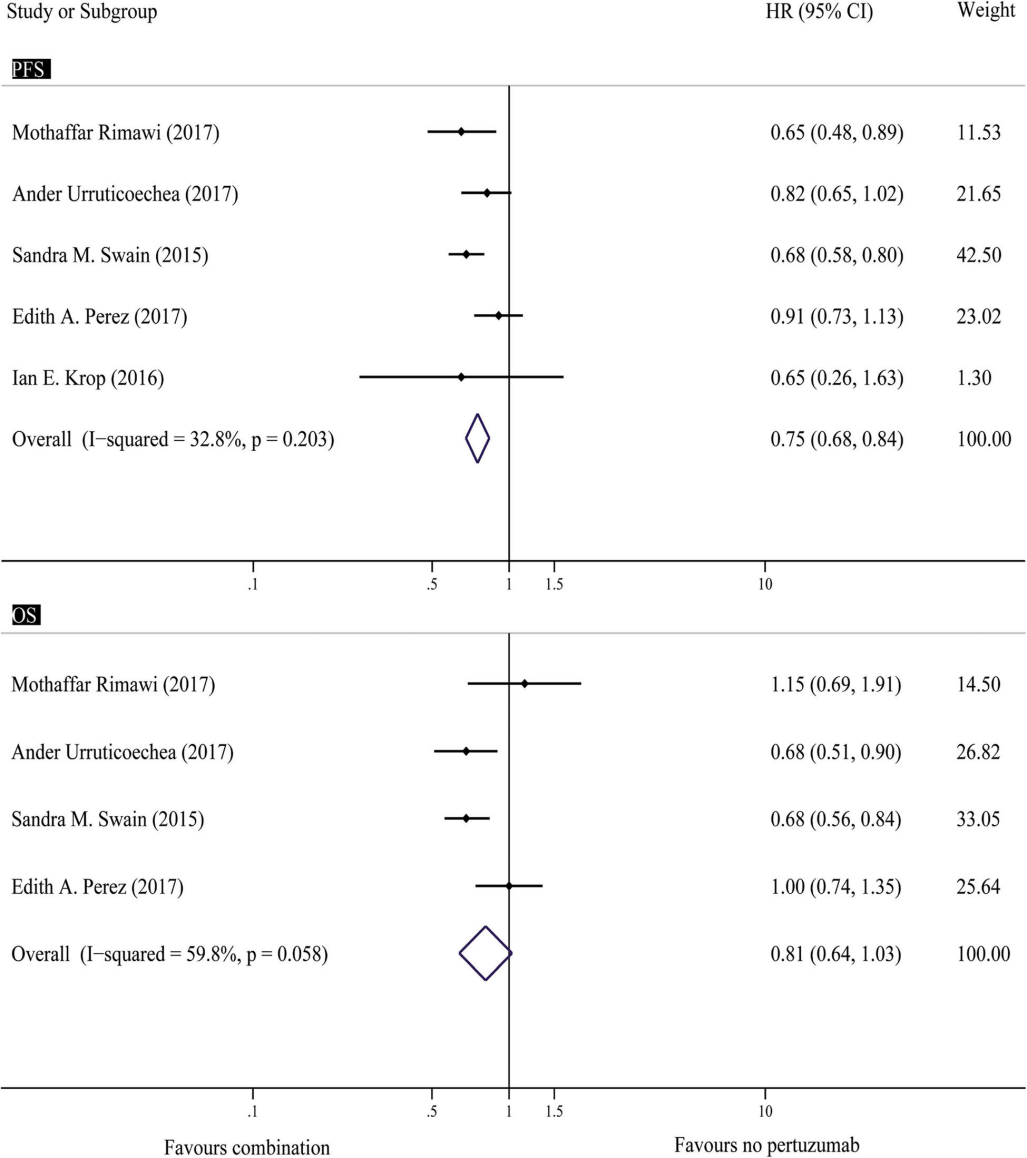
Forest plots of PFS and OS: combination of pertuzumab with HER2 inhibitors versus HER2-targeted therapies without pertuzumab for patients with HER2+ breast cancer. PFS = progression free survival, OS = overall survival, HER2 = human epidermal growth factor receptor 2, HR = hazard ratio

Thirteen trials reported the median PFS [9, 14, 18–21, 26–30, 32, 33], and four of these trials also reported OS [9, 18, 19, 21]. The robust pooled results using a fixed-effects model demonstrated that H + P might stabilize diseases and prolong the survival of HER2+ MBC. The hazard ratio was 0.75 (95% CI, 0.68–0.84; *P* < 0.001) (Fig. 3), which indicated that H + P significantly improved the median PFS in patients with HER2+ MBC. Low statistical heterogeneity among the included studies was noted (I^2^ = 32.8%; *P* = 0.203) in the PFS analysis (Fig. 3). We found no evidence of publication bias in any of the analyses using Begg’s test (*P* = 1.00) and Egger’s test (*P* = 0.974).

Regarding OS, there was a trend towards better OS but that it did not reach statistical significance (HRs = 0.81; 95% CI, 0.64–1.03; *p* = 0.082) (Fig. 3). No significant heterogeneity was observed (I2 = 59.8%; *P* = 0.058) (Fig. 3). The sensitivity analysis revealed an estimated HR of 0.71 (95% CI, 0.61–0.84) after removing the study conducted by Edith A. Perez. We also found no evidence of publication bias in any of the analyses using Begg’s test (*P* = 0.308) and Egger’s test (*P* = 0.216).

### Secondary outcomes

#### Relative risk of adverse reactions

**Fig. 4:**
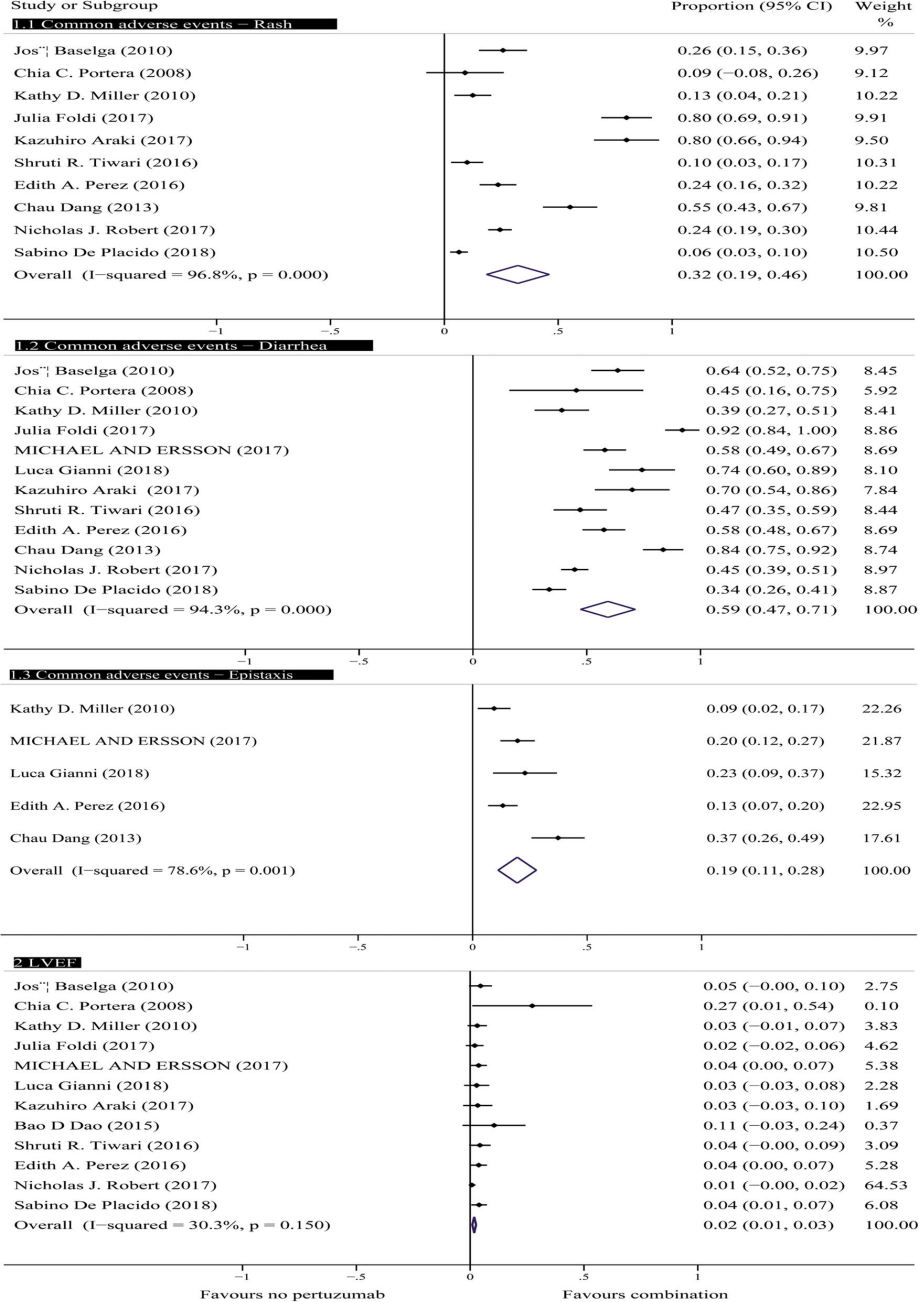
Forest plots of common adverse events and cardiotoxicity events in single-arm studies: combination of pertuzumab with HER2 inhibitors for patients with HER2+ breast cancer. HER2 = human epidermal growth factor receptor 2

**Fig. 5:**
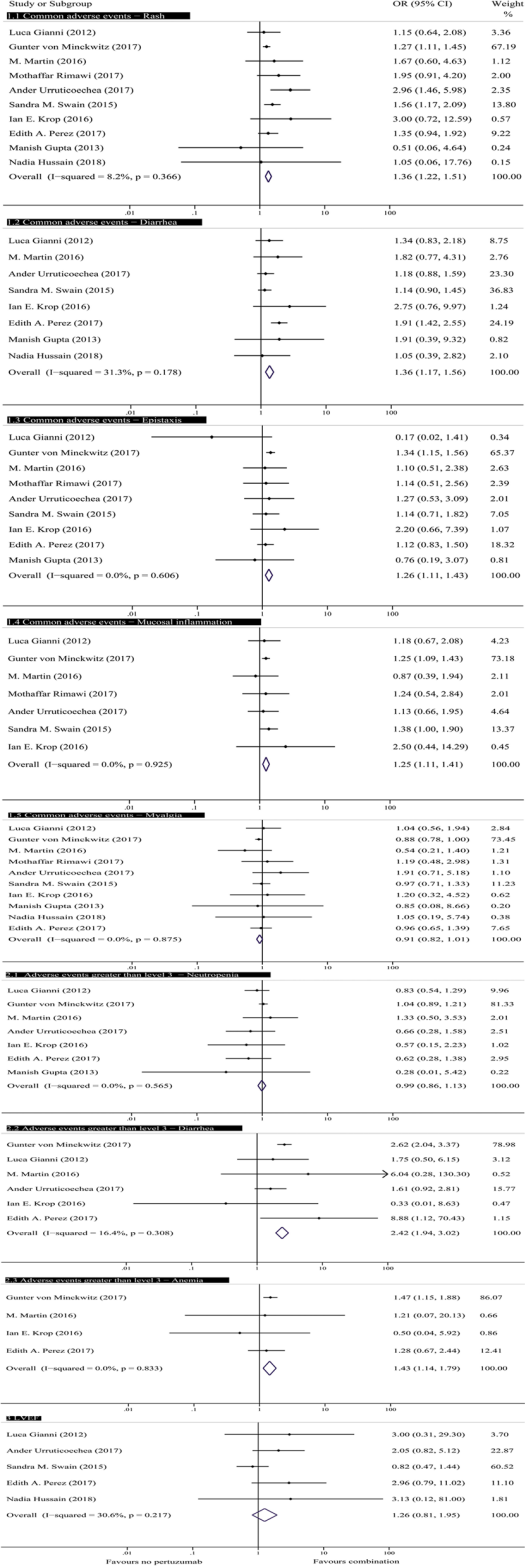
Forest plots of common adverse events, grade ≥ 3 adverse events and cardiotoxicity events in controlled studies: combination of pertuzumab with HER2 inhibitors versus HER2-targeted therapies without pertuzumab for patients with HER2+ breast cancer. HER2 = human epidermal growth factor receptor 2, OR = odds ratio

We recorded and evaluated the AEs in all 26 trials, and the most common all-grade AEs were rash, diarrhea, myalgia, epistaxis, and mucosal inflammation. We calculated the overall rate and 95% CI for some adverse reactions in the single-arm trials using a random effects model (Fig. 4). The rates ranged from 6 to 80% for rash, 34 to 92% for diarrhea, and 9 to 37% for epistaxis. The pooled absolute rates for rash, diarrhea, and epistaxis were 0.32 (95% CI, 0.19–0.46), 0.59 (95% CI, 0.47–0.71), and 0.19 (95% CI, 0.11–0.28), respectively. The sensitivity analysis showed that the pooled absolute rates for rash, diarrhea, and epistaxis were 0.8 (95% CI, 0.5–0.11), 0.41 (95% CI, 0.37–0.45), and 0.15 (95% CI, 0.11–0.18) after removing the studies conducted by José Baselga, Julia Foldi, Kazuhiro Araki, Edith A. Perez, Chau Dang, and Nicholas J. Robert. The analysis using a fixed-effects model of AEs in the controlled trials showed that the H + P group was associated with a significantly higher incidence of all-grade rash (OR = 1.36; 95% CI, 1.22–1.51; *P* < 0.001), diarrhea (OR = 1.36; 95% CI, 1.17–1.56; P < 0.001), epistaxis (OR = 1.26; 95% CI, 1.11–1.43; P < 0.001), and mucosal inflammation (OR = 1.25; 95% CI, 1.11–1.41; P < 0.001) compared with the H group. Interestingly, a tendency toward a significantly reduced incidence of myalgia was found in the H + P group (OR = 0.91; 95% CI, 0.81–1.01; *P* = 0.065). The analysis of most common all-grade AEs of H + P indicated that pertuzumab played a prominent role in the incidences of rash, diarrhea, epistaxis, myalgia, and mucosal inflammation (Fig. 5).

Among AEs of grade ≥ 3, three common AEs were neutropenia, diarrhea, and anemia. The rates for diarrhea ranged from 0.016 to 0.14, and the pooled absolute rate for diarrhea was 0.5 (95% CI, 0.4–0.7). In the controlled trials, the rates of diarrhea and anemia in the experimental group were significantly higher than those in the controlled group [(OR = 2.42; 95% CI, 1.94–3.02; *P* = 0.0001) and (OR = 1.43, 95% CI, 1.14–1.79, *P* = 0.002), respectively]. A significant difference was not observed in neutropenia (OR = 0.99, 95% CI, 0.86–1.13, *P* = 0.814) (Fig. 5).

#### Cardiac toxicity

The data for an LVEF decline < 50% or more than 10% from baseline obtained in 15 trials were analyzed. In all the studies, the LVEF was assessed at baseline and then every 3 months. The percentage of patients who experienced cardiac toxicity ranged from 0.002 to 0.27, and the pooled absolute rate for cardiac toxicity was 0.02 (95% CI, 0.01–0.03) (Fig. 4). In the controlled trials, cardiac toxicity was analyzed using a fixed-effects model, and the results showed that H + P did not increase the incidence of LVEF compared with the effect of H (OR = 1.26; 95% CI, 0.81–1.95; *P* = 0.309) (Fig. 5).

## Discussion

In this meta-analysis, we evaluated the efficacy and safety of H + P versus H for the treatment of patients with HER2+ breast cancer in (neo)adjuvant settings. The development of the first HER2-targeted therapy, trastuzumab, transformed (and significantly improved) the traditional remedies and induced AEs in the treatment of HER2+ breast cancer patients, which led to its initial approval in 1998. Despite these advances, the resistance to and severe toxicity of trastuzumab forced the development of additional anti-HER2 targeted therapies and the continuous exploration of combinatorial-targeted strategies. The development of new targeted agents, such as pertuzumab and T-DM1, revolutionized the therapeutic strategy of HER2+ breast cancer patients in clinical settings. Pertuzumab in combination with trastuzumab and docetaxel for the treatment of patients with HER2+ breast cancer has been approved by the Food and Drug Administration. T-DM1, a complex agent that combines the mechanisms of trastuzumab and maytansine, minimizes toxicity by selectively delivering the cytotoxic agent to tumor cells, thereby minimizing systemic exposure. The research prospects of the combination of pertuzumab with T-DM1 are well worth exploring. Randomized controlled trials investigating the combination of pertuzumab and T-DM1 for the treatment of breast cancer have been published in recent years [11, 13, 20, 21]. Pertuzumab-based dual anti-HER2 therapies have been widely used in the clinic, and thus, many retrospective trials are included in our study. To our knowledge, this systematic review and meta-analysis constitutes the first investigation of the benefit of H + P (pertuzumab plus trastuzumab or trastuzumab emtansine) versus H (trastuzumab or trastuzumab emtansine) and involves the first subgroup analysis conducted with respect to HR.

We observed that HER2+ breast cancer patients with a mixed HR status (positive or negative) benefited from H + P therapy in terms of pCR, PFS, and OS, regardless of the choice of chemotherapy.

In the neoadjuvant phase, the analysis of pCR (absolute difference = 11.0%; OR = 1.33; 95% CI, 1.08–1.63; *P* = 0.006) (Fig. 2a and b) showed that HER2+ breast cancer patients receiving pertuzumab achieved a greater benefit from H + P compared with that achieved from H. Peter Beitsch et al. reported a higher pCR rate than that obtained in other studies [10], and his study outcome showed that the pCR in the H + P group was higher than that in the H group (57.0 and 40.0%, respectively), with an absolute difference of 17.0%. A network meta-analysis conducted by Aiko Nagayama et al. that compared H + P with H also showed a significant difference in the pCR (OR = 2.29; 95% CI, 1.02–5.02; *P* = 0.02) [36]. A randomized controlled trial (NeoSphere) evaluated the efficacy of three treatment groups (group H + P, group H, and group P) [15]. This study showed that patients given H + P had a significantly improved pCR compared with those given H (45.8 and 29.0%, respectively), patients given P received the lowest pCR (24.0%). Currently, due to the lack of research on pertuzumab monotherapy, the only clinical trial (NeoSphere) involving pertuzumab monotherapy was analyzed in our research.

In metastatic settings, the H + P treatment of patients with HER2+ demonstrated significant benefits on PFS (HRs = 0.75; 95% CI, 0.68–0.84; *P* < 0.00001) (Fig. 3). This result indicated that H + P has a clear tendency to prolong survival. Unfortunately, statistical significance was not observed in the OS analysis (HRs = 0.81; 95% CI, 0.64–1.03; *P* = 0.082) (Fig. 3). However, we found that the efficacy of group H + P was superior to that of group H by analyzing the OS/PFS results and trended towards better OS which did not reach statistical significance. Further larger scale, well-designed RCTs are needed to identify this trend. The similar results presented in the CLEOPATRA study, a phase III study that included 808 patients with HER2+ MBC, were randomized to pertuzumab + trastuzumab + docetaxel or trastuzumab + docetaxel + placebo. In this study, the comparison of H + P and H revealed that survival was prolonged by 6.3 months. The difference in PFS was significant (HRs = 0.69, 95% CI, 0.58–0.81; *P* < 0.001), and a significant benefit in OS was observed in the patients allocated to the combined treatment group compared with those assigned to the control group (HRs = 0.66; 95% CI, 0.52–0.84; P < 0.001) [9].

Our subgroup analysis showed that H + P and H exerted different impacts on pCR outcomes according to the HR status in the neoadjuvant phase. This analysis demonstrated that the benefit from H + P was more evident in HR- than in HR+, with distinct increases of 78.0 and 45.0% in the absolute rate of pCR in the single-arm trials (P < 0.001), respectively (Fig. 2a). In contrast, a significant difference was not observed in patients with HER2+/HR+ breast cancer (OR = 1.37; 95% CI, 0.88–2.13; *P* = 0.165) or HER2+/HR- breast cancer (OR = 1.37; 95% CI, 0.91–2.07; *P* = 0.123) in controlled trials (Fig. 2b). Although similar outcomes were obtained from the comparison between the HR+ group and the HR- group, the clinical advantage from H + P is more significant in HR- than in HR+, with absolute increases of 17.0 and 9.0%, respectively. Our result was consistent with those obtained in other studies investigating the effects of combined therapy on HER2+ tumors. Gianni L et al. reported that H + P yielded higher PCR rates in HR−/HER2+ breast cancer compared with those achieved in HR+/HER2+ breast cancer (63.2 and 26.0%, respectively) [15], and M. Martin et al. reported a 36.5% improvement in the outcomes of pCR after H + P therapy in the comparison of HR−/HER2+ and HR+/HER2+ breast cancer patients. Thus, we suggest that H + P could be considered a beneficial therapeutic opportunity for patients with HER2+ breast cancer and a negative HR status. The biological mechanisms underlying the different effects according to HR status are unclear, but HR expression has been associated with anti-HER2 drug resistance in preclinical and clinical models, possibly due to cross-talk inhibition between growth-promoting pathways [37, 38].

Regarding the safety profile, the incidence of all-grade AEs, including rash, diarrhea, epistaxis, and mucosal inflammation, was significantly higher among HER2+ patients treated with H + P than among those treated with H. Interestingly, a downward trend in the incidence of myalgia was observed (OR = 0.91; 95% CI, 0.82–1.01; *P* = 0.072) (Fig. 5). Among AEs of grade ≥ 3, only diarrhea and anemia were significantly more frequent in the H + P group than in the H group, and the incidence of other AEs was not significantly aggravated (Fig. 5). In the PHEREXA trial, the highest risk of severe diarrhea was observed in the H + P group compared with that in the H group (16.2% versus 10.1%), with a significant difference of 6.1%. In the NeoSphere trial, regardless of the all-grade AEs (rash, diarrhea, and mucosal inflammation) or AEs of grade ≥ 3 (diarrhea), the risk of H + P group was higher than that of H group and P group, and the risk of P group was the lowest among the three groups. Gastrointestinal toxicity showed a strong relationship with pertuzumab treatment. Previous studies have shown that the proper functioning of the gastrointestinal tract relies on the expression of HER2 receptors in many vital structures [39], such as epithelial cells and enteric nervous system neurons [40]. Pertuzumab might act on the receptors of these normal cells and interfere with their functions, leading to gastrointestinal toxicity. Rash was the most common side effect of targeted therapies. The occurrence of rash appears to be related to the mechanism through which pertuzumab acts on the HER2 receptors of cells, similar to the mechanism associated with the occurrence of diarrhea. EGFR is the major HER/ErbB receptor expressed on human keratinocytes [41], and HER2 heterodimerizes with EGFR and ErbB3 [42]. Hence, some functional EGFR–HER2 interactions likely occur in skin, and these are likely amenable to blockade by pertuzumab. Nonetheless, future studies are needed to more clearly elucidate the mechanism of pertuzumab. Many times, these typically toxicities of therapies in clinical practice are higher than those in clinical trials due to careful selection of patients with good performance status, good organ function and excellent health otherwise. We also confirmed this statement by consulting clinicians. We found that these adverse reactions are quite common for targeted therapies, and the safety profiles of particular targeted agents are well known by breast cancer patients, which helps to reduce or even prevent the risk of some AEs. However, we must attach importance to the risk of toxicity of anti-HER2 dual block therapies to maximize patient benefit. In the clinic, doctors may adjust the dose according to the individual needs of the patients with the aim of reducing the occurrence of AEs or take measures to prevent these effects.

Our study also analyzed heart safety profiles because the HER2 signaling pathway plays an important role in cardiac physiology [43]. The outcomes observed in 17 single-arm trials showed that HER2-targeted therapies including pertuzumab are harmful to heart safety (Fig. 4). However, our analysis of controlled trials revealed no increased risk of cardiac toxicity associated with the addition of pertuzumab to anti-HER2 therapies (Fig. 5), which is consistent with the results of a previous study conducted by Antonis Valachis et al. [44].

The addition of pertuzumab to HER2-targeted monotherapy reduced the risk of disease recurrence and death among patients who had developed drug resistance due to long-term treatment with single HER2 inhibitors, and the incidence of serious adverse reactions caused by the use of high-dose single HER2 inhibitors was decreased. The comparison of the benefits between the two treatment groups revealed that the H + P groups still showed a strong advantage, regardless of whether they were combined with chemotherapy (palbociclib, fulvestrant, vinorelbine, taxane, eribulin, doxorubicin+cyclophosphamide, carboplatin, paclitaxel, fluorouracil+epirubicin+cyclophosphamide, and aromatase inhibitors). Additionally, we summarized the administered dosages of H + P included in this study. Among the included trials, the most common administrations were pertuzumab or placebo (a loading dose of 840 mg administered intravenously followed by a dose of 420 mg administered intravenously every 3 weeks) and trastuzumab (a loading dose of 8 mg/kg administered intravenously followed by a dose of 6 mg/kg administered intravenously every 3 weeks) or T-DM1 (3.6 mg/kg). Our results not only enhance the prominent role of pertuzumab added to dual anti-HER2 targeted therapies in the (neo)adjuvant treatment of HER2+ breast cancer but also alleviated some of the confusion regarding the benefit of adding pertuzumab to HER2 therapies and effectively revealed the importance of individualized therapy.

This review has several strengths and limitations. First, to our knowledge, this study constitutes the systematic review and meta-analysis aiming to investigate the benefit of anti-HER2 dual blockade (pertuzumab plus trastuzumab or trastuzumab emtansine) compared with that of monotherapy (trastuzumab or trastuzumab emtansine) and includes the first subgroup analyses conducted with respect to the HR status. Second, our study included a sufficiently large sample, which increases the statistical power of the evaluation of the effect of the combination treatment. Third, we also assessed the effects of dual-blockage treatment on a subpopulation of patients with different HR statuses. Fourth, we evaluated the efficacy and safety of the treatment of patients with HER2+ breast cancer at various stages. Several limitations include the following. First, several of the controlled trials lacked complete data and included nonrandomized controlled trials, and fewer samples were included in the single-arm trials. Second, the calculations were based on published study results and presented clinical trials rather than individual patient data, which might generate biases.

## Conclusions

In conclusion, the results of this systematic review and meta-analysis provide the first opportunity to compare the efficacy and safety of HER2 inhibitors with (H + P) or without pertuzumab (H) for patients with HER2+ breast cancer. Our meta-analysis confirms that H + P is superior to H in the (neo)adjuvant treatment of HER2+ breast cancer, and increase the risk of acceptable and tolerable toxicity (rash, diarrhea, epistaxis, mucosal inflammation, and anemia). Based on the subgroup analysis of pCR, H + P is a correct choice for the treatment of patients with HER2+/HR- breast cancer. The combined application of pertuzumab and HER2-targeted drugs is thus promising and potent.

## Data Availability

All data are available in this manuscript.

## Abbreviations

AEs: Adverse events
CI: Confidence interval
H + P: HER2 inhibitors + pertuzumab ± chemotherapy
H: HER2 inhibitors ± chemotherapy
HER2: Human epidermal growth factor receptor 2
HR-: Hormone receptor negative
HR+: Hormone receptor positive
HRs: Hazard ratios
LVEF: Left ventricular ejection fraction
OR: Odds ratio
OS: Overall survival
pCR: Pathologic complete response
PFS: Progression-free survival
RCT: Randomized controlled trial
T-DM1: Trastuzumab emtansine
